# Influence of the practice of physical exercise and healthy eating on the Vigour of university lectures

**DOI:** 10.3389/fspor.2023.1228668

**Published:** 2023-11-27

**Authors:** Montserrat Monserrat Hernández, Ángeles Arjona Garrido, Juan Carlos Checa Olmos, Antonio Casimiro Andújar, Eva María Artés Rodríguez, Darío Salguero García

**Affiliations:** ^1^ Social Anthropology, Department Geography, History and Humanities, University of Almería, Almería, Spain; ^2^ Sociology, Department Geography, History and Humanities, University of Almería, Almería, Spain; ^3^ Physical Education, Department Educational Sciences, University of Almería, Almería, Spain; ^4^ Statistics, Department of Math, University of Almería, Almería, Spain; ^5^ Psychology, Department of Psychology, University of Almería, Almería, Spain

**Keywords:** physical exercise, nutrition, vigour, university professors, health

## Abstract

**Introduction:**

Vigour at work is characterized by high levels of energy and high desire to make an effort at work. This article is the result of a research carried out with university teachers in Spain whose main objective is to show what type and frequency of physical exercise and diet influence Vigour.

**Methods:**

The sample consisted of 121 subjects, 62% of whom were women and 37.2% men. A questionnaire was administered to collect information on sociodemographic data, physical exercise habits, eating habits and Vigour at work. Cross-tabulations of the dimensions of Vigour with sex, age and type of contract offered were performed. Spearman correlations and Correspondence Analysis are also carried out to provide information on the intensity and type of relationships between the Vigour dimensions. Finally, the influence of the frequency of physical exercise and diet on Total Vigour is investigated.

**Results:**

The results show that the relationships between the dimensions are very strong (sig = 0.001). In addition, the practice of moderate-high intensity physical exercise and maintaining a good adherence to the Mediterranean Diet is related to high levels of Total Vigour (F = 7.955; sig = 0.006). As for the influence of the sociodemographic variables used, significant differences were only observed in the Physical Strength dimension for sex (*X*^2^ = 6.173; *p* = 0.046) and age (*X*^2^ = 9.449; *p* = 0.051) and, with respect to the type of contract, in Emotional Energy (*X*^2^ = 19.487; *p* < 0.001).

**Discusión and conclusions:**

The main conclusions of our study show that practicing physical exercise of medium-high intensity four hours or more per week and a high adherence to the MD is more related to high Vigour levels than just eating well or just practicing exercise. And more studies are needed on the influence of sociodemographic variables on Vigour and its different dimensions.

## 1. Introduction

Vigour at work is characterised by high levels of energy and passion in the execution of work, with the intention that both the process (attitude) and the product (result) respond to the expectations created. Vigour is understood as a positive interaction between physical strength, emotional and cognitive energy, which will favour an increase in work performance (1, 2). Physical strength refers to one's own capabilities and the effort made to efficiently execute the tasks assigned. Emotional energy brings together the interpersonal components of emotional intelligence, i.e., healthy relationships with others: sympathy, empathy, assertiveness, understanding and support for others. Finally, cognitive capacity relates to mental agility, reasoning and fluency of thought. The aim is to achieve a balance between the three dimensions.

Research on the influence of Vigour on work effectiveness is still limited. However, Shirom (3) uses Fredrickson and Losada's (4) Broaden and Built Theory of positive emotions or B&B as a basis for demonstrating the consequences that Vigour at work produces for employees, explaining that positive affectivity facilitates cognitive flexibility by helping to create resources such as adaptive coping strategies.

The literature is grouped in different lines of work that provide some basis for suggesting that a Vigorous employee is likely to have a relevant impact on work outcomes, either through the atmosphere created, perceived effectiveness or even connection to work in an engaged way (5–8). The ways of measuring work effectiveness can be multiple: individual and collective (6, 9), or through exogenous or endogenous factors (10–12); even from the point of view of the subject's own satisfaction with the perceived work and its results (13).

Although the above measurements are in line with the work environment, we must not forget that people also interact in other environments (family, friends, leisure, etc.), so that the way we perform in each of these scenarios influences the rest (14).

Similarly, lifestyle can influence the general sense of well-being and thus work (9). In this respect, both physical exercise and diet are important variables to consider (15). Regarding physical activity, studies show that adequate and continuous physical exercise offers work-related psychological benefits, such as reduced stress, anxiety, perceived self-efficacy, etc. (16–18). Other research concludes that a sense of self-fulfilment and self-esteem (feeling strong, perceiving an acceptable physique, etc.) also has a positive impact on other aspects of life, including work (19, 20).

With regard to diet and its relationship with well-being at work, the literature is not very extensive, focusing mainly on its impact on "generalised fatigue" or *Burnout* (21–23). Several studies show that there is a relationship between the practice of certain nutritional patterns such as the Mediterranean Diet (MD), Mediterranean Diet-DASH Intervention for Neurodegenerative Delay (MIND), vegan diets, etc., and physical and emotional fatigue (24–26).

Consequently, there is a need for research into the combined influence of physical exercise and healthy eating on workers' feelings of vigour. For this reason, in this study we want to analyse the influence that a healthy lifestyle, mainly the practice of physical exercise and an adequate diet, has on the vigour at work (exploratory analysis). The scope of application is the teaching staff of Andalusian public universities (Spain).

Our starting hypotheses were: (1) gender (male) and type of contract (government official-staff) will positively and significantly influence the Vigour of university professors; (2) practicing physical exercise (any intensity) regularly (minimum 3 days a week) will be positively and significantly related to scores in total Vigour and its dimensions; (3) high levels of adherence to the MD will be positively and significantly related to high values of total Vigour and its dimensions; (4) practicing physical exercise regularly (minimum 3 days a week) and maintaining a high adherence to the MD will offer better results in total Vigour and its dimensions than diet alone or physical exercise alone.

## 2. Material and method

### 2.1. Participants

Participants were obtained through non-probability sampling using the *snowball* method, as the sample is limited to a small subset of the population. After defining the participation program describing the invitation process, the Andalusian public universities were contacted (through the corresponding vice-rectorships) (to guarantee the diversity of contacts throughout Andalusia) to disseminate the mailing of suggestion for participation, and after obtaining the initial contacts, access to other contacts was requested. This was done until a representative sample of the Spanish university teaching staff was obtained (27) with a confidence interval of 95% and a margin of error of 8%.In the end, the study population consisted of a total of 121 subjects, 62.8% female and 37.2% male. Table 1 shows a more detailed description of the sample by age group and type of contract.

**Table 1:** Descriptive data of the sample (sex, age groups, type of contract).

		*N* (%)
Sex	Women	75 (62,0)
	Men	45 (37,2)
Age groups	Between 20 and 40 years old	27 (22,3)
	Between 41 and 50 years old	39 (32,2)
	Between 51 and 60	51 (42,1)
	Over 60 years old	4 (3,3)
Type of contract	Permanent contract	71 (58,7)
	Non-fixed contract	50 (41,3)

Own elaboration.

### 2.2. Instrument and procedure

The information was obtained by administering an anonymous survey through the Limesurvey platform. The fieldwork lasted from December 2022 to February 2023. The variables that make up the questionnaire are grouped into several blocks: socio-demographic, physical exercise habits, eating habits and Vigour at work.

Physical exercise practice was measured by: hours per week spent exercising and the intensity level of the sessions, with medium-low intensity considered to be those where the heart rate does not exceed 70% of their maximum heart rate, or, subjectively, breathing is not too fast and they can easily follow a conversation during exercise; medium-high intensity is considered to be those activities that exceed 70% of their maximum heart rate: The exercise intensity will be obtained by Tanaka's formula, which is obtained by multiplying the age by 0.7 and subtracting the result from 208. For example, for a 44 year old person it would be: [208—(44 × 0.7)] = 177 p/m. In this case, 70% (124 p/m) would be applied to this figure. Or subjectively, breathing is not very fast and can easily follow a conversation during the activity; medium-high intensity is considered to be those activities that exceed 70% of their maximum heart rate (for those who could measure it) or, subjectively, those who observe that their breathing is difficult, fast and can hardly speak while they are doing the activity.

To measure the frequency of food consumption we used the CFCGA (Cuestionario de Frecuencia de Consumo de Grupos), based on an Exchange System and validated for the Spanish population (28). For dietary habits we used the Mediterranean Diet Score (MDS) (29).

Vigour at work was measured through the Shirom-Melamed Vigour Scale (2), based on three subscales that reflect the theoretical dimensions of Vigour: physical strength, cognitive Vigour and emotional energy. They are interpreted by means of a Likert-type scale, ranging from 1 (never or almost never) to 7 (always or almost always), on how they feel in various situations.

In the analysis, the variables gender, age and type of contract are introduced, distinguishing between permanent and non-permanent. The former are civil servants and permanent contract teachers, while the latter are part-time contract teachers.

The Vigour dimensions were recoded into low, medium and high, dividing the total score by three and obtaining a reduced scale for each ranging from 1 to 3. The variable of frequency of physical exercise was recoded as follows: no exercise (0), two hours or less (1), between 2 and 4 h (2), more than 4 h (3). Vigour levels were recoded into low (1), medium (2) and high (3). As for adherence to the Mediterranean diet, it is low when it is equal to or less than 9 points and optimal when it is higher than 9.

The study was conducted according to the guidelines of the Declaration of Helsinki and Ley Orgánica de Protección de Datos y Garantía de Derechos Digitales (LOPDGDD 3/2018) that regulates the processing of data of both minors and adults. In addition, it was approved by the Bioethics Committee of the University of Almeria (UALBIO2023/005).

### 2.3. Statistical analysis

Firstly, a descriptive analysis was carried out to observe the general data of the sample on: Vigour, exercise and diet. After the frequencies and cross-tabulations, correlations were made to observe the relationship of weekly practice with type of exercise intensity (low-medium and medium-high) with the dimensions that define Vigour. And, in order to obtain information regarding the relationships between the dimensions of Vigour and Total Vigour, a Correspondence Analysis was carried out. Since the results showed that exercise offered significant correlations with all the dimensions of Vigour, except with the emotional dimension, and low-moderate intensity exercise was only related to the emotional dimension, it was decided to carry out a two-factor ANOVA to see if adding the dietary factor (adherence to DM) to moderate-high intensity physical exercise would lead to different results.

All statistical analyses were performed using the SPSS-27 statistical software.

## 3. Results

Table 2 shows the descriptive statistics of the different variables analysed (physical exercise intensity, MD Adherence and Vigour dimensions). With regard to the practice of physical exercise, both low-moderate and moderate-intense intensity, we observe an M of 0.51 and 0.77, respectively, which implies a practice of 2 h or less per week. On the other hand, there is similarity in the means of the dimensions that make up Vigour (M of 2.413, 2.487 and 2.545), meaning an "average" score for the surveyed group in general. With regard to adherence to DM, the mean does not reach the score considered "good adherence" (more than 9 points) and none of the respondents achieves a score of 14 (maximum level).

**Table 2:** Descriptive statistics of the variables used in the analysis.

	Minimum	Maximum	Mean (DT)
Moderate FE practice	0.00	3.00	0.512 (0.797)
Intense FE practice	0.00	3.00	0.776 (0.978)
Physical Strength	1.00	3.00	2.413 (0.703)
Emotional energy	1.00	3.00	2.487 (0.620)
Cognitive vivacity	1.00	3.00	2.545 (0.632)
MDS	1.00	13.00	8.661 (2.249)

Own elaboration.

### 3.1. Relationship between Vigour and sex, age group and type of contract

Table 3 shows that there are no significant differences in Total Vigour scores with respect to gender, age or type of contract. However, the data reflect a trend towards "high" scores in both sexes and in the 41–50 age group.

**Table 3:** Results in Total Vigour by sex, age group and type of contract. *N* (%).

	M (DT)	Sex*		Age**				Type of contract***	
Total Vigour	2.51 (0.59)	Women *N* (%)	Men *N* (%)	20–40	41–50	51–60	>60	Fixed	Not fixed
Under		5 (6.0)	1 (2.4)	3 (11.1)	2 (5.1)	1 (1.9)	0 (0)	4 (5.6)	2 (4)
Medium		34 (43.1)	13 (30.9)	15 (55.5)	9 (23.0)	21 (41.17)	2 (50)	29 (40.8)	18 (36)
High		40 (50.9)	28 (66.6)	9 (33.3)	28 (71.7)	29 (56.8)	2 (50)	38 (53.5)	30 (60)

Own elaboration.

* *X*^2^ = 3.148; *P* = 0.207.

** *X*^2^ = 12.054; *P* = 0.061.

*** *X*^2^ = 0.554; *P* = 0.758.

In Table 4 there are also no significant differences with respect to gender, age group and type of contract in terms of cognitive vivacity. The data again show a trend towards "high" scores in both sexes and in the 41–50 age group with respect to cognitive vivacity.

**Table 4:** Results in cognitive vivacity (vigour dimension) according to sex, age group and type of contract. *N* (%).

	M (DT)	Sex*		Age**				Type of contract***	
Cognitive vivacity	2.54 (0.63)	Woman	Man	20–40	41–50	51–60	>60	Fixed	Not fixed
Under		7 (8.8)	2 (4.7)	3 (11.1)	2 (5.1)	4 (7.8)	0	6 (8.4)	3 (6)
Medium		26 (32.9)	11 (26.1)	11 (40.7)	12 (30.7)	13 (25.4)	1 (25)	22 (30.9)	15 (30)
High		46 (58.2)	29 (69.0)	13 (48.1)	25 (64.1)	34 (66.6)	3 (75)	43 (60.5)	32 (64)

Own elaboration.

* *X*^2^ = 1.542; *P* = 0.462.

** *X*^2^ = 3.615; *P* = 0.730.

*** *X*^2^ = 0.302; *P* = 0.860.

In terms of physical strength, there are differences in terms of sex and age group, with men and the 41–60 age group having the highest percentages in the "high level" (see Table 5).

**Table 5:** Results in physical strength (vigour dimension) according to sex, age group and type of contract. *N* (%).

	M (DT)	Sex*		Age**				Type of contract***	
Physical strength	2,41 (0,70)	Women	Men	20–40	41–50	51–60	>60	Fixed	Not fixed
Under		12 (16)	3 (6.6)	6 (22.2)	1 (2.5)	8 (15.6)	0 (0)	12 (16.9)	3 (6)
Medium		31 (41.3)	10 (22.2)	13 (48.1)	12 (30.7)	14 (27.4)	2 (50)	23 (32.3)	18 (36)
High		36 (48)	29 (64.4)	8 (29.6)	26 (66.6)	29 (56.8)	2 (50)	36 (50.7)	29 (58)

Own elaboration.

* *X*^2^ = 6.173; *P* = 0.046.

** *X*^2^ = 9.449; *P* = 0.051.

*** *X*^2^ = 3.216; *P* = 0.200.

Emotional Energy (Table 6) shows significant differences in terms of the type of contract, with teachers on permanent contracts showing the highest percentages in the "high level".

**Table 6:** Emotional energy (vigour dimension) scores by gender, age group and type of contract. *N* (%).

	M (DT)	Sex*		Age**				Type of contract***	
Emotional energy	2,48 (0.68)	Woman	Man	20–40	41–50	51–60	>60	Fixed	Not fixed
Under		6 (8)	2 (4.4)	4 (14.8)	3 (7.6)	1 (1.9)	0 (0)	4 (5.6)	4 (8)
Medium		27 (36)	19 (42.2)	8 (29.6)	15 (38.4)	21 (41.1)	2 (50)	16 (22.53)	30 (60)
High		46 (61.3)	21 (46.6)	15 (55.5)	21 (53.8)	29 (56.8)	2 (50)	51 (71.83)	16 (32)

Own elaboration.

* *X*^2^ = 1.551; *P* = 0.461.

** *X*^2^ = 5.590; *P* = 0.470.

*** *X*^2^ = 19.487; *P* < 0.001.

### 3.2. Relationship between physical exercise intensity and Vigour dimensions

Spearman's correlation analysis (Table 7) showed significant and positive relationships between the practice of moderate-intense physical exercise and the Physical Strength and Cognitive Vivacity dimensions. This has a significant impact on Total Vigour directly (exercise-Total Vigour). However, the practice of low-moderate intensity physical exercise correlates only with the Emotional Energy dimension, not directly affecting Total Vigour, but indirectly, through Emotional Energy.

**Table 7:** Correlational analysis between the different dimensions of vigour and the practice of physical exercise.

	Physical Strength	Cognitive Vivacity	Emotional energy	Total Vigour
Moderate-high intensity physical exercise	0.329**	0.244**	−0.057	0.242**
Low-moderate intensity physical exercise	−0.073-	−0.015	0.249**	−0.006
Physical Strength	1	0.594**	0.336**	0.767**
Cognitive Vivacity	0.594**	1	0.442**	0.737**
Emotional energy	0.336**	0.442**	1	0.538**
Full vigour	0.767**	0.737**	0.538**	1

* Correlation is significant at the 0.005 level (bilateral).

** Correlation is significant at the 0.01 level (bilateral).

Own elaboration.

**Figure 1:**
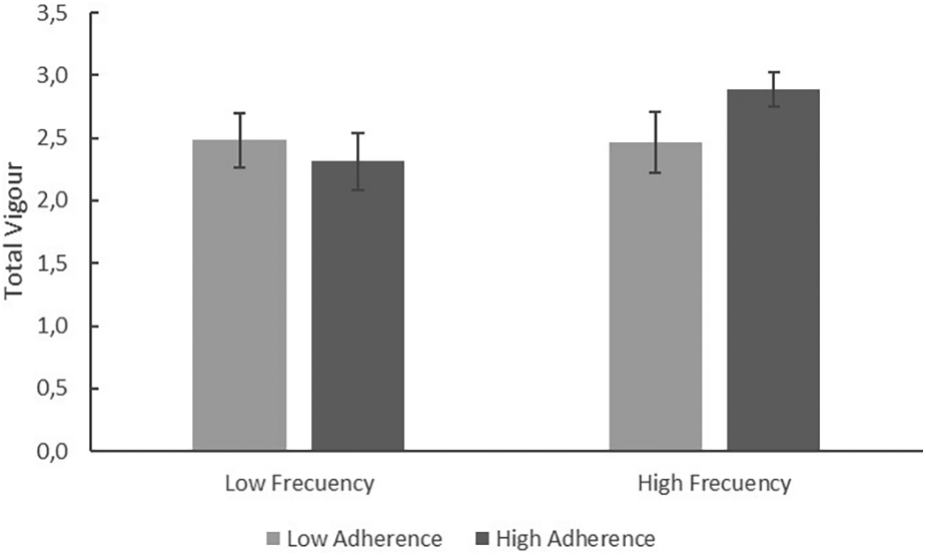
Impact on total vigour of the relationship between frequency of moderate-high intensity PE and adherence to DM. Source: Own elaboration.

The Correspondence Analysis graphs are shown in Figure 1. In the case of Total Vigour and Physical Strength (sig < 0.001) all scores are represented in both rows and columns, due to the high scores obtained in terms of dimension contribution: Total Vigour (low = 0.520; medium = 0.870 and high = 0.988); Physical Strength (low = 0.743; medium = 0.651 and high = 0.979). However, the negative scores in the dimension regarding "low level" and "medium level" of both Total Vigour and Physical Strength place them in opposition to the "high level" appearing on the positive side of the graph.

Relating both dimensions, it can be seen that they follow the same trend, and the higher the level of both, the greater the confluence (see Figure 1). This shows that there is a relationship between Physical Strength and Vigour the higher the scores on both dimensions.

In the Correspondence Analysis Total Vigour-Cognitive Vivacity (sig < 0.001) only the values "high level" and "low level" will be taken into account because only these two scores are optimal in the contribution of the dimension: Total Vigour (low = 0.958; mean = 0.224; high = 0.434); Cognitive Vivacity (low = 0.979; mean = 0.001; high = 0.461). Furthermore, the graph also shows that "low level" is placed in opposition to "high level" in both variables and, as in the previous analysis, the higher the level in both, the greater the confluence of the points, the higher the Cognitive Vivacity, the higher the Total Vigour.

And, in the Correspondence Analysis Total Vigour-Emotional Energy (sig < 0.001) also only the values "high level" and "low level" will be taken into account due to the dimension contribution scores in both: Total Vigour (low = 0.999; medium = 0.054; high = 0.590); Emotional Energy (low = 1.00; medium = 0.233; high = 0.540). So the "high level" conflates more strongly with Total Vigour compared to the other dimensions.

### 3.3. Influence of moderate-intense physical exercise and DM on Vigour

Since the practice of low-moderate intensity physical exercise was only directly related to Emotional Energy and, the homoscedasticity tests of the two-factor ANOVA (low-moderate intensity PE and Mediterranean Diet) tested the null hypothesis that the dependent variable is equal between groups (sig = 0.252), it was decided to go deeper into the analysis of moderate-high intensity physical exercise.

The practice of moderate-high intensity physical exercise is directly and positively related to Total Vigour; hence, the next step was to test whether the frequency of exercise, together with the consumption of foods considered healthy, within the DM recommendations, leads to an improvement in the practice of such exercise. For this, we performed a two-factor ANOVA independent measures 2 × 2 with Total Vigour as the dependent variable and frequency of PE practice and adherence to the MD as intersubject factors. Means follow a normal distribution (*p* > 0.001) (see Table 8).

**Table 8:** Descriptive statistics of the intervening variables in the ANOVA. Total Vigour as dependent variable.

Frequency of EF Moderate-intense^a^	Adherence to DM^b^	Media	Standard Deviation	N
1	0	2.481	0.509	27
1	1	2.315	0.661	38
	Total	2.384	0.604	65
2	0	2.466	0.628	30
2	1	2.884	0.325	26
	Total	2.660	0.548	56
Total	0	2.473	0.570	57
	1	2.546	0.615	64
	Total	2.512	0.593	121

^a^ Less than 4 h per week (value 1); more than 4 h per week (value 2).

^b^ No adherence (MDS scores below 9) (value 0); Yes adherence (MDS scores above 9) (value 1).

Own elaboration.

Levene's test shows that the null hypothesis that the error variance of the dependent variable is equal between groups does not hold. Since *p* = 14.493; gl3 = 3; gl2 = 117; with sig < 0.001.

As can be seen in Table 9 and Figure 1, adherence to DM alone is not influential on Total Vigour, but the practice of moderate-intense physical exercise is. However, if moderate-high intensity physical exercise is performed and good adherence to DM is maintained, Total Vigour levels increase exponentially.

**Table 9:** Tests of inter-subject effects. Dependent variable Total Vigour.

Origin	Type III sum of squares	gl	Root mean square	F	Sig	Partial Eta squared
Corrected model	5.160^a^	3	1.720	5.428	0.002	0.122
Intersection	762.078	1	762.078	2,405.14	<0.001	0.954
Frequency EF moderate-intense	2.271	1	2.271	7.168	0.008	0.058
Adherence to DM	0.471	1	.471	1.486	0.225	0.013
Frequency EF*Adherence DM	2.520	1	2.520	7.955	0.006	0.064
Error	37.072	117	0.317			
Total	806.000	121				
Total adjusted	42.231	120				

Own elaboration.

^a^ *R*^2^ = 0.122; adjusted *R*^2^ = 0.100.

* The interaction between both variables.

## 4. Discussion

The main objective of this research was to study the frequency and intensity of physical exercise that provides the greatest benefits for the vigour of university lecturers. After the analysis carried out, it was found that the practice of physical exercise with a higher weekly frequency and moderate-high intensity (>70% of maximum heart rate) offers better results than lower intensities. Other studies confirm that moderate-high intensity exercise, such as power work, has been associated with: (1) control of depression and other negative emotional states (30); (2) physical and physiological changes that lead to a more acceptable body image and better self-concept and self-esteem (31); (3) effective achievement of personal goals, thanks to perseverance, which favours a sense of emotional well-being (32).

All these consequences of increased physical-sports practice can be transferred to the workplace as shown by Watanave et al. (33) who indicate that higher levels of work resources are associated with higher Vigour in those who practice physical exercise. Even, adding physical exercise programs or active rest in the same workspace improves the results in Total Vigour (34) or in some of its dimensions (35–38). However, guidelines on frequency and intensity in these studies remain unspecified.

Although the most significant results on Vigour derive from moderate-high intensity physical exercise, it is interesting to note the influence of low-moderate intensity exercise on Emotional Energy. This contribution, added to the fact that the Correspondence Analysis shows a high relationship between high Emotional Energy scores and Total Vigour, highlights the importance of lower intensity physical activities such as yoga, pilates, walking, etc., which favour moments of relaxation, activate the parasympathetic nervous system and provide balance for dealing with stressful situations and improve the quality of sleep. In this way, these benefits help to carry out daily activities in a more conscious way, which can offer positive results in terms of work performance (39–41) and their perception of mental health (38, 42).

Evidently, there are other factors external to physical exercise that influence the teachers' sense of well-being, such as a healthy diet that can have an impact on Total Vigour, thus making it possible to establish possible recommendations on lifestyle habits. Some studies relate healthy eating habits to dimensions related to cognitive performance, emotional state and feelings of physical strength (43, 44).

In this way, it was observed that by adding the practice of more than 4 h of moderate-high intensity physical exercise per week together with a diet based on DM, the results on Total Vigour increase exponentially. However, there are no significant differences in terms of following a MD pattern in those who do less than 4 h a week, which underlines the importance of continuity in the practice (more benefits with greater frequency of practice). Possibly, in this aspect we can observe two interesting factors: on the one hand, to corroborate that the practice of physical exercise is the most influential variable and, on the other, that the greater the practice of exercise, the greater the need to eat correctly in order to continue to perform both in sport (45) and in other scenarios of life (including work).

All of this must be "just right", as excessive exercise can lead to overtraining syndrome, understood as a state of physical and mental exhaustion associated with excessive physical exercise, both in intensity and duration, which has a negative impact on work activity and energy when carrying out daily activities (46, 47). In addition, an adequate diet adapted to the type of training (among others) is essential, as a preventive factor for overtraining, etc. (48, 49).

As for the influence on Vigour of variables such as gender, age and type of contract, no significant differences were found unlike other studies that show differences respect to gender (50) or respect to contract type (51). Further research is needed. In our case, in the Physical Strength dimension, men between 41 and 60 years of age did show higher scores than women and the rest of the age groups. On the other hand, it was also shown that those with permanent contracts had higher levels of Emotional Energy. These data, although they do not directly influence Total Vigour, should be taken into account because there are strong correlations and correspondence relationships between the dimensions. Furthermore, age, sex and type of contract are variables that can be modified little or not at all by the subject, but exercise and eating habits can (52, 53).

With this research we also wanted to provide additional information and demonstrate the possible benefits of adding a healthy diet to the Total Vigour results, thus establishing possible recommendations on lifestyle habits. In this way, it was observed that by combining the practice of moderate-high intensity physical exercise for more than 4 h a week with a diet based on DM, the results on Total Vigour increase exponentially, but there are no significant differences in terms of following a DM pattern and not following it in those who do less than 4 h a week. Possibly, in this aspect we can observe two interesting factors, on the one hand, to corroborate that the practice of physical exercise is the most influential variable and, on the other hand, that the more physical exercise is practised, the greater the need to eat correctly in order to continue to perform both in the field of sport and in the other scenarios of life (including work). In this way, we avoid, in part, developing overtraining syndrome, understood as a state of physical and mental exhaustion associated with excessive physical exercise in terms of both intensity and duration, and then its subsequent transfer to work. In this regard, we refer to the research carried out on the influence of overtraining on energy when carrying out daily activities, which has been observed to have a negative influence (46). And, furthermore, the importance of an adequate diet (among others) as a preventive factor for overtraining: adequate intake of macronutrients, adequacy of micronutrients, adaptations according to the type of training, etc. (48, 49).

### 4.1. Limitations

The study has limiting factors to consider. The first is that the sampling is not random; the *snowball* strategy was used to collect the data, a tactic also used in other studies on the same type of target sample) (54, 55).

The second concerns observational errors (56) related to the impossibility of obtaining all the influential variables, so that we cannot establish with certainty the state of the question.

Thirdly, the possibility of measurement errors, since there are disparate percentages in terms of gender, which may be due to the degree of motivation in the self-administration of the questionnaire. Therefore, further work is needed to reduce these sampling errors and to increase the variables, for example, physical exercise and/or socio-demographic variables.

Fourth, with respect to the healthy eating variable, we only considered adherence to DM, ignoring the possible existence of other dietary patterns that could influence the results. Therefore, other patterns should be taken into account in future research.

## 5. Conclusions

As it has been demonstrated in the literature, the consequences of Vigour are behavioral, cognitive and emotional and, therefore, it plays an important role at work and in life in general.

With the present research, we wanted to add more information to the literature so that it can serve as support for organizations when implementing proposals to improve the health of their workers, since knowing regulation strategies that demonstrate their significant influence on Vigour will be of special importance and will give security to those who develop them. Thus, institutional actions may suggest the implementation of programs that promote knowledge of the situation, physical training and appropriate nutritional recommendations that allow them to be able to develop them in their day-to-day work. In general, the main conclusions obtained from our study subjects have been:

(1) Practicing physical exercise minimum 4 h a week and at a medium-high frequency is beneficial for Vigour.
(2) Following the MD shows no relationship with Vigour.
(3) Practicing physical exercise of medium-high intensity four hours or more per week and a high adherence to the MD is more related to high Vigour levels than just eating well or just practicing exercise.
(4) More studies are needed on the influence of sociodemographic variables on Vigour and its different dimensions.

All the above, we consider the present study to have a high explanatory and justifying value, since it offers information on a field that has hardly been studied. These data show that the different life processes, as well as job stability, are considered to be influential in professional life. However, given that certain situations are not the result of their own decisions (gender, age and type of contract), we consider it interesting that, on the part of university institutions, teachers are advised and encouraged to carry out activities that can be controlled by themselves and, therefore, offer benefits to their general and occupational health, such as physical exercise and a healthy diet.

## Data Availability

The original contributions presented in the study are included in the article/Supplementary Material, further inquiries can be directed to the corresponding author.

## References

[1] Blomme RJ Kodden B Beasley-Suffolk A. Leadership theories and the concept of work engagement: creating a conceptual framework for management implications and research. J Manag Organ. (2015) 21(2):125–44. 10.1017/jmo.2014.71

[2] Shirom A. Feeling vigorous at work? The construct of vigor and the study of positive affect in organizations. In: Ganster DC Perrewe PL, editors. Emotional and physiological processes and positive intervention strategies (Research in occupational stress and well being), 3. Bingley: Emerald Group Publishing Limited (2004). p. 135–64. 10.1016/S1479-3555(03)03004-X

[3] Shirom A. Vigor as a positive affect at work: conceptualizing vigor, its relations with related constructs, and its antecedents and consequences. Rev Gen Psychol. (2011) 15(1):50–64. 10.1037/a0021853

[4] Fredrickson BL Losada MF. Positive affect and the complex dynamics of human flourishing. Am Psychol. (2005) 60:678–86. 10.1037/0003-066X.60.7.678 16221001 PMC3126111

[5] Arriaza I. Relación entre bienestar laboral, capital psicológico y vigor en una muestra de trabajadores de distintos sectores [Dissertation/thesis]. Jaén: University of Jaén (2022).

[6] Campos ML Velasco C Araya JP. Adaptación y validación de escalas de medición en el trabajo. Part 2: collective effectiveness. Inf Technol. (2020) 31(6):43–52. 10.4067/S0718-07642020000600043

[7] Khan H Rehmat M Butt TH Farooqi S Asim J. Impact of transformational leadership on work performance, burnout and social loafing: a mediation model. Futur Bus J. (2020) 6:1–13. 10.1186/s43093-020-00043-8

[8] Tafvelin S Nielsen K von Thiele Schwarz U Stenling A. Leading well is a matter of resources: leader vigour and peer support augments the relationship between transformational leadership and burnout. Work Stress. (2019) 33(2):156–72. 10.1080/02678373.2018.1513961

[9] López P Gallegos V. Leadership practices and the mediating role of collective efficacy in teachers’ job satisfaction. Estudios Pedagógicos (Valdivia). (2014) 40(1):163–78. 10.4067/S0718-07052014000100010

[10] Cabrera A Belmonte JL González ME Cevallos M. Design, validation and application of a questionnaire to measure the influence of exogenous factors on the effectiveness of reverse learning. Psychol Soc Educ. (2020) 12(1):1–16. 10.7821/naer.2021.1.574

[11] Hadipour E Mohammad Khani K Mohammad Davoudi A. Identify and analyze the effective barriers on organizational vitality in government organizations. Iran J Educ Soc. (2022) 8(1):97–108. 10.22034/ijes.2021.537484.1144

[12] Jovari B. Organizational vigour creation model in universities. Int J Manag Bus Res. (2023) 7(1):28–49. 10.5281/zenodo.8217674

[13] Cuesta M Aguado M. Self-perception of health, quality of life and psychological well-being in a sample of older adults. Revista Española de Comunicación en Salud. (2019) 10(1):21–9. 10.20318/recs.2019.3993

[14] Peiró JM Montesa D Soriano A. Systematic review of research on the relationships between well-being and job performance in ibero-America. Anuario Internacional de Revisiones en Psicología. (2021) 1:95–121. 10.14635/REVPSY.0.5

[15] Entrena Durán F. Globalización, identidad social y hábitos alimentarios. Revista de Ciencias Sociales (Cr). (2008) 1(119):27–38. 10.15517/rcs.v0i119.10783

[16] Estrada P Vázquez E Gáleas Á Ortega I Serrano MD Acosta J. Psychological benefits of physical activity at work in an educational center. Challenges. (2016) 30:203–6. 10.47197/retos.v0i30.50254

[17] Gómez R Grimaldi M Bernal A Fernández J. Physical activity practice and its relationship with job satisfaction in a food organization. J Sports Econom Manag. (2016) 2(6):85–98. Available at: http://hdl.handle.net/11441/66098

[18] Obando IA Calero S Carpio P Fernández A. Effect of physical activities on the decrease of work stress. Revista Cubana de Medicina General Integral. (2017) 33(3):345–51. Available at: https://www.medigraphic.com/cgi-bin/new/resumen.cgi?IDARTICULO=79152

[19] Azofeifa C. Review of the benefits of physical exercise intensity and modalities on psychological stress. Think Motion. (2018) 16(1):1–21. 10.15517/pensarmov.v16i1.30335

[20] Barroso P. Study of the effects of a physical exercise programme in the workplace on the health of workers [Dissertation/thesis]. Madrid: Universidad Europea de Madrid (2017).

[21] Blanco LS Lamprea LT Patiño B Sánchez D. Afectación de síndrome de burnout en las organizaciones [Dissertation/thesis]. Bogotá: Universidad Politécnica Grancolombiano (2020).

[22] Bonet J Parrado E Barahona A Capdevila L. Development and application of a combined evaluation system of physical exercise, nutrition and psychological variables in young university students. Apunts. Medicina de L'Esport. (2016) 51(191):75–83. 10.17979/sportis.2018.4.1.2062

[23] Gómez C Palma S. A global, updated and critical view of the role of sugar in our diet. Nutrición Hospitalaria. (2013) 28:1–4. Available at: http://scielo.isciii.es/scielo.php?script=sci_arttext&pid=S0212-16112013001000001&lng=en&nrm=iso

[24] Ramada N. Mediterranean Diet versus vegetarian diet in depressive disorder [Dissertation/thesis]. Cantabria: Universidad Europea del Atlántico (2021).

[25] Rodríguez N. Comparison of different dietary patterns for the prevention of major depressive disorder [Dissertation/thesis]. Cantabria: Universidad Europea del Atlántico (2022).

[26] Saiz P Wang Y Saiz JG Fernández VG Sánchez C. Lifestyle, adherence to the Mediterranean diet, anthropometric characteristics in a group of university students with health deficiencies. Rev Esp Nutr Comun. (2017) 23(2):1–11.

[27] Gobierno de España, Ministerio de Universidades. Estadística de personal de las universidades (2022). Available at: https://www.universidades.gob.es/wp-content/uploads/2023/02/NOTA_EPU_21_22.pdf

[28] Goni L Aray M Martínez A Cuervo M. Validation of a food group consumption frequency questionnaire based on an exchange system. Hosp Nutr. (2016) 33(6):1391–9. 10.20960/nh.800 28000471

[29] Trichopoulou A Costacou T Bamia C Trichopoulous D. Adherence to a Mediterranean diet and survival in a Greek population. N Engl J Med. (2003) 348(26):2599–608. 10.1056/NEJMoa025039 12826634

[30] Guarin A. Human resiliency [Dissertation/thesis]. Colombia: Universidad de los Andes (2016).

[31] Góngora L Giraldo D. Proposal of continuous aerobic training and HIIT for people with obesity [Dissertation/thesis]. Colombia: Universidad del Valle (2017).

[32] Arenas Á Calderón J. Effect of a high intensity interval training (HIIT) programme on body mass Index, aerobic capacity, explosive strength and speed in schoolchildren aged 15 to 18 years [Dissertation/thesis]. Manizales: Universidad Autónoma de Manizales (2020).

[33] Watanabe K Otsuka Y Inoue A Sakurai K Ui A Nakata A. Interrelationships between job resources, vigor, exercise habit, and serum lipids in Japanese employees: a multiple group path analysis using medical checkup data. Int J Behav Med. (2016) 23:410–7. 10.1007/s12529-015-9516-9 26475033

[34] Michishita R Jiang Y Ariyoshi D Yoshida M Moriyama H Obata Y La introducción de un programa de descanso activo por parte de las unidades de trabajo mejoró el vigor en el lugar de trabajo y el presentismo entre los trabajadores. Revista de Medicina Ocupacional y Ambiental. (2017) 59(12):1140–7. 10.1097/JOM.0000000000001121

[35] Albulescu P Macsinga I Rusu A Sulea C Bodnaru A Tulbure BT. "Give me a break!" A systematic review and meta-analysis on the efficacy of micro-breaks for increasing well-being and performance. PLoS One. (2022) 17(8):e0272460. 10.1371/journal.pone.0272460 36044424 PMC9432722

[36] Jindo T Kai Y Kitano N Tsunoda K Nagamatsu T Arao T. Relación del ejercicio en el lugar de trabajo con el compromiso laboral y el malestar psicológico en los empleados: un estudio transversal del estudio MYLS. Informes de Medicina Preventive. (2020) 17:101030. 10.1016/j.pmedr.2019.101030

[37] Luger T Maher CG Rieger MA Steinhilber B. Programas de descanso laboral para prevenir síntomas y trastornos musculoesqueléticos en trabajadores sanos. Base de datos Cochrane de revisiones sistemáticas, (7) (2019). 10.1002/14651858.CD012886.pub2 PMC6646952 31334564

[38] Lyubykh Z Gulseren D Premji Z Wingate TG Deng C Bélanger LJ Role of work breaks in well-being and performance: a systematic review and future research agenda. J Occup Health Psychol. (2022) 27(5):470. 10.1037/ocp0000337 35980721

[39] Amórtegui C Calderón N Jiménez J Lozano E Segura J. Fomento de prácticas de autocuidado para prevenir el estrés laboral, mediante pausas activas en el área de venta de la empresa Bimbo: Politécnica Grancolombiano (2021).

[40] Cossio M. El aprendizaje del yoga como herramienta para el control del malestar laboral [Dissertation/thesis]. Madrid: Universidad Pontifica Comillas (2021).

[41] Melero A. Yoga para ejecutivos: Técnicas eficaces de relajación para mejorar el rendimiento en el trabajo. Profit Editorial (2015).

[42] Asztalos M Bourdeaudhuij I Cardon G. The relationship between physical activity and mental health varies across activity intensity levels and dimensions of mental health among women and men. Public Health Nutr. (2009) 13(8):1207–14. 10.1017/S1368980009992825 20018121

[43] Leedo E Beck AM Astrup A Lassen AD. The effectiveness of healthy meals at work on reaction time, mood and dietary intake: a randomised cross-over study in daytime and shift workers at an university hospital. Br J Nutr. (2017) 118(2):121–9. 10.1017/S000711451700191X 28820084

[44] Firth J Gangwisch JE Borsini A Wootton RE Mayer EA. Food and mood: how do diet and nutrition affect mental wellbeing? Br Med J. (2020) 369:2382. 10.1136/bmj.m2382 PMC7322666 32601102

[45] Panão I Carraça EV. Effects of exercise motivations on body image and eating habits/behaviours: a systematic review. Nutr Diet. (2020) 77(1):41–59. 10.1111/1747-0080.12575 31449357

[46] Cárdenas D Conde J Perales JC. Fatigue as a subjective motivational state. Andalusian J Sports Med. (2017) 10(1):31–41. 10.1016/j.ramd.2016.04.001

[47] Lichtenstein MB Hinze CJ Emborg B Thomsen F Hemmingsen SD. Compulsive exercise: links, risks and challenges faced. Psychol Res Behav Manag. (2017) 10:85–95. 10.2147/PRBM.S113093 28435339 PMC5386595

[48] Koch G Cancino J Roco Á Jorquera C Aguilera R Hernández M. Heart rate control, energy intake and sleep quality in classical dancers. Finlay J. (2018) 8(4):284–90. Available at: http://scielo.sld.cu/scielo.php?script=sci_arttext&pid=S2221-24342018000400006&lng=es&nrm=iso

[49] Sánchez F Vaamonde A. Overtraining and sport from a psychological perspective: state of the art. Revista de Psicología Aplicada al Deporte y al Ejercicio Físico. (2017) 2(2):1–12. 10.5093/rpadef2017a8

[50] Sonnentag S y Niessen C. Mantenerse vigoroso hasta que termine el trabajo: el papel del vigor rasgo, las experiencias laborales específicas del día y la recuperación. Revista de Psicología Ocupacional y Organizacional. (2008) 81(3):435–58. 10.1348/096317908X310256

[51] Levecque K Rigolle F De Beuckelaer A Mortier A. Phd students and vigour: on bursting with energy, feeling fit and being enthusiastic about work. Ecoom Briefs. (2019) 21:1–5. Available at: https://www.researchgate.net/profile/Anneleen-Mortier/publication/361207988_PhD_students_and_vigour_on_bursting_with_energy_feeling_fit_and_being_enthusiastic_about_work/links/62a2f0d1a3fe3e3df86c4b37/PhD-students-and-vigour-on-bursting-with-energy-feeling-fit-and-being-enthusiastic-about-work.pdf

[52] Lavie CJ Ozemek C Carbone S Katzmarzyk PT Blair SN. Sedentary behavior, exercise, and cardiovascular health. Circ Res. (2019) 124(5):799–815. 10.1161/CIRCRESAHA.118.312669 30817262

[53] Pearce M Garcia L Abbas A Strain T Schuch FB Golubic R Association between physical activity and risk of depression: a systematic review and meta-analysis. JAMA Psychiatry. (2022) 79(6):550–59. 10.1001/jamapsychiatry.2022.0609 35416941 PMC9008579

[54] Ariz M. Profesores adscriptores en enseñanza Media [Dissertation/thesis]. Uruguay: Universidad ORT (2020).

[55] Arroyo P. Perfil del profesorado universitario inclusivo: experiencia del alumnado durante el confinamiento [Dissertation/thesis]. Castelló: Universitat Jaume I (2021).

[56] Groves RM. Survey errors and survey cost. John Wiley and Sons (1989).

